# The superior accuracy of a novel method in total hip wear calculations following radiographic measurement

**DOI:** 10.1186/s12891-021-04964-5

**Published:** 2022-02-09

**Authors:** Kazutaka So, Koji Goto, Atsushi Kawaguchi, Yutaka Kuroda, Shuichi Matsuda

**Affiliations:** 1 Department of Orthopaedic Surgery, Osaka Red Cross Hospital, 5-30 Fudegasaki-cho, Tennoji-ku, Osaka city, Osaka, 543-8555 Japan; 2 Department of Orthopaedic Surgery, Kyoto University, 54 Shogoin Kawahara-cho, Sakyo-ku, Kyoto City, Kyoto 606-8507 Japan; 3 Education and Research Center for Community Medicine, Faculty of Medicine, Saga University, 5-1-1 Nabeshima, Saga City, Saga 849-8501 Japan

## Abstract

**Background:**

Polyethylene wear is one of the major concerns of orthopedic surgeons. However, there is no standardized calculation method for the wear rate following radiographic measurement. The purpose of this study was to propose a novel method of wear calculation and to compare its accuracy with a representative conventional method.

**Methods:**

Relative position of the center of the femoral head to that of the cup progresses in one direction following arthroplasty surgery because of bedding-in and wear. We predetermined the amount of bedding-in, wear rate, and random error in measuring the head center position in a 2-dimensional plane. We calculated the wear rate using the head center coordinates over a certain number of measurement periods using a representative conventional method and our novel method. The conventional method consisted of transforming vector data into scalars and conducting a least-squares method. The least-squares method was directly applied to each component of the vector in the novel method. We evaluated the accuracy of these methods by comparing the expected value for the wear rate with their predetermined true values.

**Results:**

If the error were limited to being random, the novel method could provide the predetermined wear rate as the calculation result. However, the conventional method could not.

**Conclusion:**

We recommend using the novel method for the wear calculation rather than the conventional method because of its mathematical accuracy.

**Supplementary Information:**

The online version contains supplementary material available at 10.1186/s12891-021-04964-5.

## Introduction

**Fig. 1:**
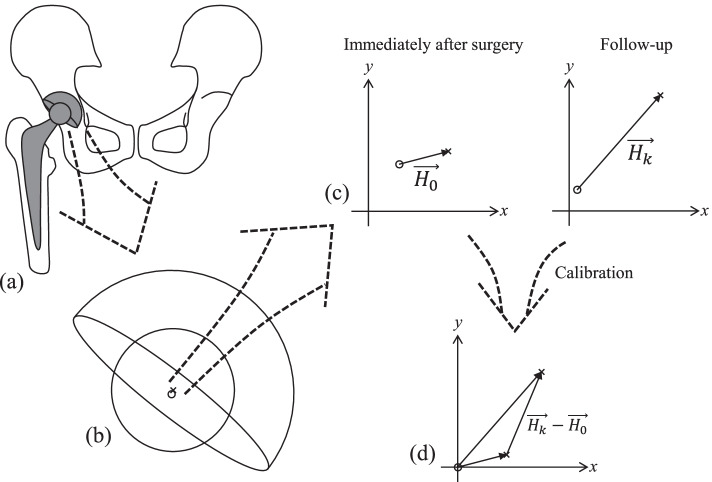
Summary of the conventional method to calculate penetration. **a** Radiographs taken immediately following the operation and at the follow-up period. **b** Detection of circular edges of the cup and head and calculation of their center positions (circle and cross) are semi-automatically performed by the measurement application. **c** Relative positions of the head center to the cup center are calculated for each radiograph ($\overrightarrow{{H}_{0}}$ and $\overrightarrow{{H}_{k}}$). **d** The head penetration during the follow-up period can be calculated as $\left|\overrightarrow{{H}_{k}}-\overrightarrow{{H}_{0}}\right|$ or $-\left|\overrightarrow{{H}_{k}}-\overrightarrow{{H}_{0}}\right|$

Progress of polyethylene wear significantly influences the long-term durability of the total hip prosthesis [1, 2]. In vivo wear can be observed as the femoral head penetration on postoperative radiographs, and numerous papers of wear analysis have been published [3–7]. There are several techniques to measure the penetration of the femoral head on radiographs after total hip arthroplasty, such as Livermore’s, the dual circle technique, and radiostereometric analysis [8, 9]. Modern measurement applications, such as Hip Analysis Suite (University of Chicago Medical Center, Chicago, Il, USA) and PolyWare (Draftware Developers, Vevay, IN, USA), measure the relative position of head center to that of cup center on radiographs [10, 11]. They then provide the head center penetration data in a scalar format, after transformation from vector (Fig. 1).

Relative position of the center of the femoral head to that of the cup is considered to progress in one direction after the arthroplasty surgery as result of bedding-in and wear. The bedding-in is considered to occur during the first several years after the operation as the result of the creep deformation of polyethylene, and the wear continues at a steady pace throughout the postoperative period as a result of friction between the head and liner [8]. Random and systematic errors are inevitable in measuring the position of the head and cup center [12–16]. Therefore, it is common in wear calculations to apply the least-squares method to multiple measured values of penetration after the bedding-in period [17]. Although the head penetration theoretically directs upward, measured penetration sometimes directs downward, due to measurement error. In this case, measurement applications provide the measurement result of penetration as $-\left|\overrightarrow{{H}_{k}}-\overrightarrow{{H}_{0}}\right|$, and such penetration is called negative wear. However, how the negative wear should be treated in the following wear calculations has not been standardized. There are several options for treating the negative wear, as used in previous papers: using it intact, ignoring, and assuming zero [18–21]. However, no study has yet evaluated the validity of each option or recommended which option is to be used.

This study had two purposes. The first was to evaluate the accuracy of the conventional method of the wear calculation. The second was to propose a new method for the wear calculation and evaluate its accuracy.

## Methods

### Predetermined conditions and notations about the head and cup center measurement

For the purpose of evaluating the accuracy of the wear calculations, a set of generalized data of the wear measurement of a single hip was prepared in this study. It was assumed that the head center penetrated postoperatively in accordance with the principle of polyethylene wear.

**Fig. 2:**
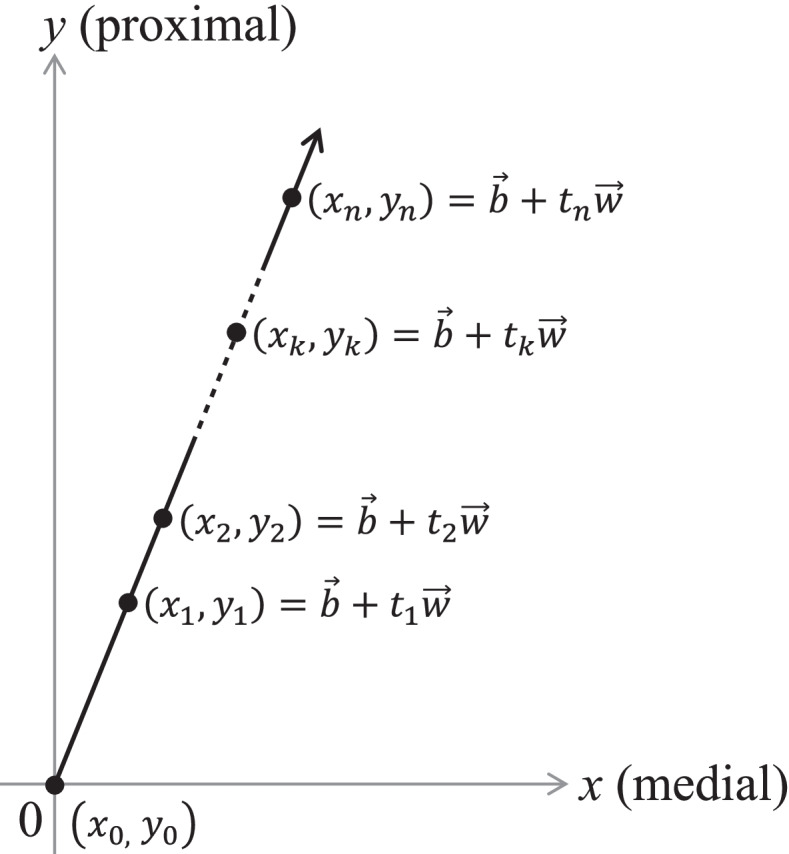
Mathematical notations about the true positions of the femoral head center. Head center coordinates relative to the cup center immediately after the operation were defined as $\left({x}_{0}, {y}_{0}\right)=(0, 0)$. Penetration of the head center by the bedding-in $\overrightarrow{b}=\left({b}_{x}, {b}_{y}\right)$ starts immediately after the operation and was presumed not to end until the first measurement period (*t*_1_). Penetration by wear starts immediately after the operation and continues at a steady pace throughout the follow-up period: $\overrightarrow{w}=\left({w}_{x}, {w}_{y}\right)$ per year

The horizontal and vertical lines were defined as the *x*- and *y*-axes, respectively, and the coordinates of the head center relative to the cup center immediately after surgery was defined as (0, 0). The medial and proximal directions were defined as positive, and the lateral and distal directions as negative. Bedding-in was defined as $\overrightarrow{b}=\left({b}_{x},{b}_{y}\right)$ and presumed to occur until the first postoperative follow-up (*t*_1_). Wear was defined to progress steadily after surgery at a rate of $\overrightarrow{w}=\left({w}_{x},{w}_{y}\right)$ per year ($\overrightarrow{b}$ and $\overrightarrow{w}$ were parallel, and ${b}_{y}>0, {w}_{y}>0$). According to these definitions, the coordinates of the head center at postoperative period *t*_*k*_ (year) could be calculated as

1 $$\left({x}_{k},{y}_{k}\right)=\overrightarrow{b}+{t}_{k}\overrightarrow{w}=\left({b}_{x}+{t}_{k}{w}_{x},{b}_{y}+{t}_{k}{w}_{y}\right),$$

where measurements of the head center positions are presumed to be performed *n* + 1 times in this study (*k* = 0, 1, 2, …, *n*, *t*_0_ = 0) (Fig. 2).

**Fig. 3:**
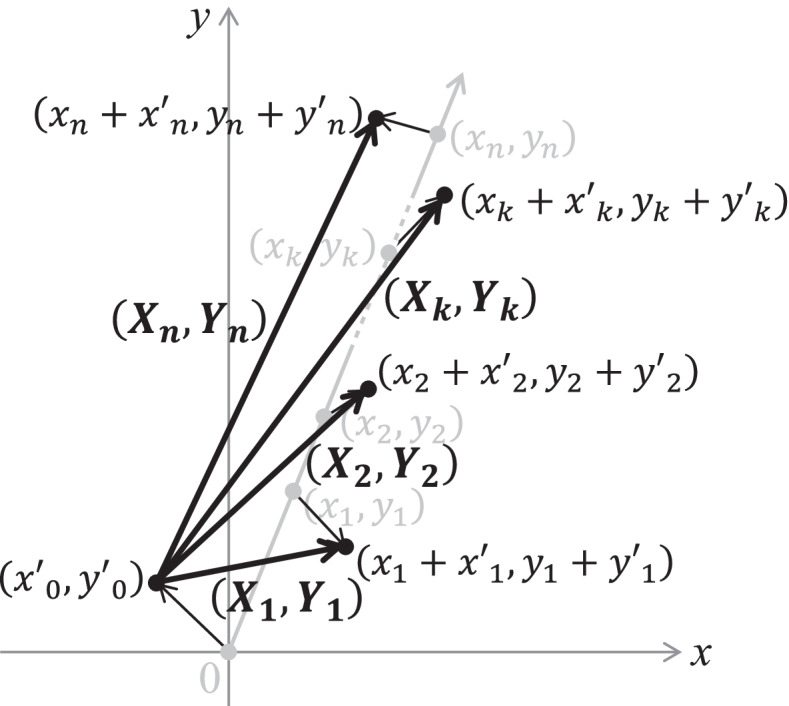
Mathematical notations about the head center positions with measurement errors. Measurement errors at postoperative period *t*_*k*_ were defined as $\left({x}_k^{\prime },{y}_k^{\prime}\right)$ (*k* = 0, 1, …, *n*), then the measured coordinates of the head center at postoperative period *t*_*k*_ were $\left({x}_k+{x}_k^{\prime },{y}_k+{y}_k^{\prime}\right)$. Penetration vector during *t*_*k*_ years was notated as $\left({X}_{k},{Y}_{k}\right)$, which were calculated as $\left({x}_k+{x}_k^{\prime }-{x}_0^{\prime },{y}_k+{y}_k^{\prime }-{y}_0^{\prime}\right)$

Errors in measuring $\left({x}_{k},{y}_{k}\right)$ were defined as $\left({x}_k^{\prime },{y}_k^{\prime}\right)$. Then, $\left({x}_k+{x}_k^{\prime },{y}_k+{y}_k^{\prime}\right)$ could be used as the coordinates measured at postoperative period *t*_*k*_. For accurate evaluation of the following wear calculations, the systematic error was presumed to be eliminated from $\left({x}_k^{\prime },{y}_k^{\prime}\right)$. Then $\left({x}_k^{\prime },{y}_k^{\prime}\right)$ represent random errors in measuring $\left({x}_{k},{y}_{k}\right)$, and

2 $$E({{x}^{\prime}}_{k})=E({{y}^{\prime}}_{k})=0,$$

where *E* is the expected value. The bedding-in and steady-state wear rate were calculated using the conventional and novel methods based on these definitions. Both calculation methods start after the penetration vector, and the measurement errors at each follow-up period were provided (Fig. 3).

3 $$\left({X}_{k},{Y}_{k}\right)=\left({x}_{k}+{x\mathrm{^{\prime}}}_{k}-{x\mathrm{^{\prime}}}_{0},{y}_{k}+{y\mathrm{^{\prime}}}_{k}-{y\mathrm{^{\prime}}}_{0}\right)$$

### Accuracy evaluation of the wear calculations using the predetermined measurement values

The accuracy of the wear calculation method was evaluated by comparing the predetermined true values of the bedding-in ($\overrightarrow{b}$) and wear rate ($\overrightarrow{w}$) with their expected values of the calculated results using $\left({X}_{k},{Y}_{k}\right)$ (*k* = 0, 1, 2, …, *n*). The calculation method could be claimed as accurate when they were consistent [22].

Meanwhile, the best-fit line for multiple points $\left({u}_{k},{v}_{k}\right)$ (*k* = 1, 2, …, *n*) is $y=ax+b$, *a* and *b* can be calculated as follows using the least-squares method [17, 23].

$$\begin{array}{l}a=\frac{n\sum_{k=1}^{n}{u}_{k}{v}_{k}-\sum_{k=1}^{n}{u}_{k}\sum_{k=1}^{n}{v}_{k}}{C}.\\ b=\frac{\sum_{k=1}^{n}{({u}_{k})}^{2}\sum_{k=1}^{n}{v}_{k}-\sum_{k=1}^{n}{u}_{k}{v}_{k}\sum_{k=1}^{n}{u}_{k}}{C}.\\ \left(C=n{\sum }_{k=1}^{n}{({u}_{k})}^{2}-\left({\sum }_{k=1}^{n}{{u}_{k}}\right)^{2}\right)\end{array}$$

These formulae can be represented more simply as follows:

4 $$a={\textstyle\sum_{k=1}^n}M_kv_k$$

5 $$b={\textstyle\sum_{k=1}^n}N_kv_k$$

where $${M}_{k}=\frac{n{u}_{k}-\sum_{l=1}^{n}{u}_{l}}{C}, {N}_{k}=\frac{\sum_{l=1}^{n}{\left({u}_{l}\right)}^{2}-{u}_{k}\sum_{l=1}^{n}{u}_{l}}{C}.$$

### Conventional method

**Fig. 4:**
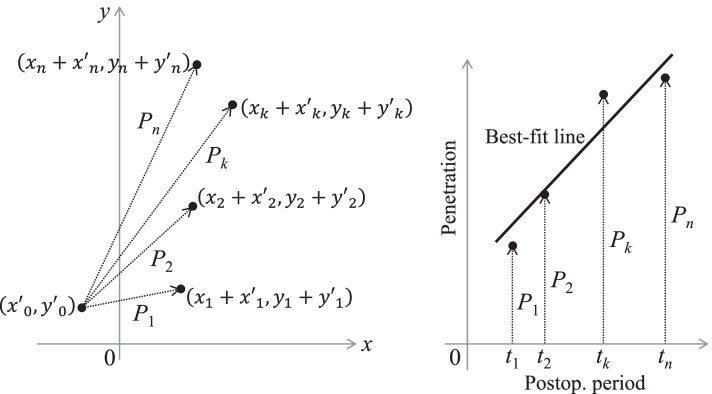
The conventional method to calculate the bedding-in and wear rate. *P*_*k*_ was calculated for each postoperative period (*t*_*k*_) using Eq. (6). The best-fit line for multiple penetration data (*t*_*k*_, *P*_*k*_) was identified using the least-squares method. The wear rate and bedding-in were indicated by the slope (*W*_*c*_) and *y*-intercept (*B*_*c*_) of the line, respectively

Conventional wear calculations start by transforming the penetration vectors with measurement errors into scalar. Negative wear was used intact in this study because it seemed the most popular option in previous studies [18–21]. Therefore, the penetration at *t*_*k*_ years $({P}_{k})$ was calculated as:

6 $${P}_{k}=sgn\left({Y}_{k}\right)\sqrt{{\left({X}_{k}\right)}^{2}+{\left({Y}_{k}\right)}^{2}},$$

where $sgn\left({Y}_{k}\right)=1$ when ${Y}_{k}\geqq 0$, and $sgn\left({Y}_{k}\right)=-1$ when ${Y}_{k}<0.$ Using the least-squares method for linear regression (Eqs. (4) and (5)), the steady-state wear rate and bedding-in (${W}_{c}$ and ${B}_{c}$) were calculated as follows (Fig. 4).

$$\begin{array}{c}W_c=\sum_{k=1}^nM_kP_k\\B_c=\sum_{k=1}^nN_kP_k\end{array}$$

$$(M_k=\frac{nt_k-\sum_{l=1}^nt_l}C,N_k=\frac{\sum_{l=1}^n\left(t_l\right)^2-t_k\sum_{l=1}^nt_l}C,C=n{\textstyle\sum_{k=1}^n}{(t_k)}^2-\left({\textstyle\sum_{k=1}^n}t_k\right)^2)$$

Therefore, we were able to conclude that the conventional method was accurate when the following equations were satisfied.

7 $$E\left({\textstyle\sum_{k=1}^n}M_kP_k\right)={\textstyle\sum_{k=1}^n}M_kE\left(P_k\right)=\left|\overrightarrow w\right|$$

8 $$E\left({\textstyle\sum_{k=1}^n}N_kP_k\right)={\textstyle\sum_{k=1}^n}N_kE\left(P_k\right)=\left|\overrightarrow b\right|$$

When all measurements were performed without an error, that is, ${P}_{k}=\left|\overrightarrow{b}+{t}_{k}\overrightarrow{w}\right|=\sqrt{{\left({x}_{k}\right)}^{2}+{\left({y}_{k}\right)}^{2}},$ the calculated results were consistent with the true values (${W}_{c}=\left|\overrightarrow{w}\right|, {B}_{c}=\left|\overrightarrow{b}\right|$). Thus,

$$\begin{array}{c}\sum_{k=1}^nM_k\sqrt{\left(x_k\right)^2+\left(y_k\right)^2}=\left|\overrightarrow w\right|\\{\textstyle\sum_{k=1}^n}N_k\sqrt{\left(x_k\right)^2+\left(y_k\right)^2}=\left|\overrightarrow b\right|\end{array}$$

Because ${M}_{k}$ and ${N}_{k}$ could take any real values depending on the measurement period

9 $$E\left({P}_{k}\right)=\sqrt{{\left({x}_{k}\right)}^{2}+{\left({y}_{k}\right)}^{2}}$$

was necessary and sufficient for Eqs. (7) and (8) to be satisfied.

### Novel method

**Fig. 5:**
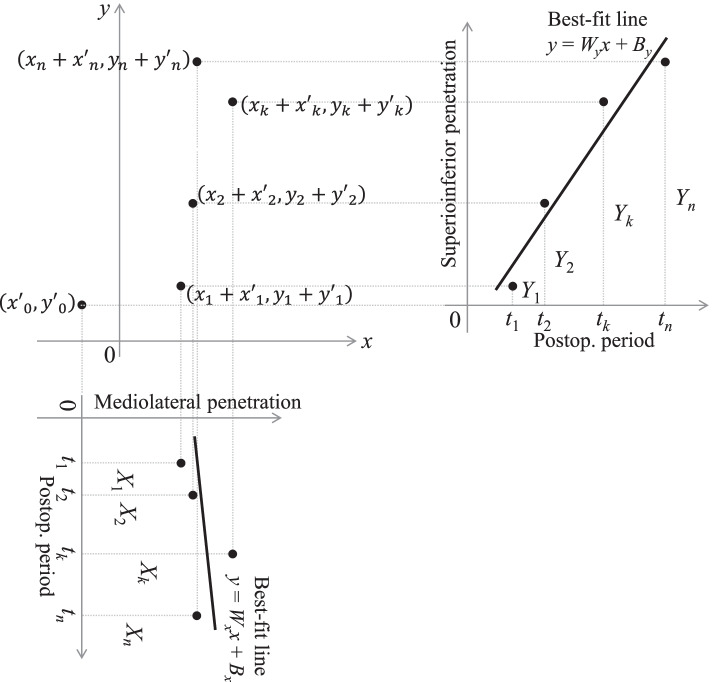
The novel method to calculate the bedding-in and wear rate. The *x* and *y* components of the penetration vector (*X*_*k*_ and *Y*_*k*_) were independently calculated for each postoperative period (*t*_*k*_). Best-fit lines for $\left({t}_{k}, {X}_{k}\right)$ and $\left({t}_{k}, {Y}_{k}\right)$ were identified separately using the least-squares method (lower left and upper right). The *x* and *y* components of the wear rate and bedding-in were indicated by the slope (*W*_*x*_, *W*_*y*_) and *y*-intercept (*B*_*x*_, *B*_*y*_) of the lines, respectively

We propose a novel calculation method in which penetration vectors are used without the transformation before the linear regression. In this method, the *x* and *y* components of the wear rate and bedding-in (*W*_*x*_, *B*_*x*_, *W*_*y*_, and *B*_*y*_) were calculated separately. Best-fit lines for $\left({t}_{k},{X}_{k}\right)$ and $\left({t}_{k},{Y}_{k}\right)$ (*k* = 1, 2, …, *n*) were respectively defined as

$$\begin{array}{c}y={W}_{x}x+{B}_{x}\\ y={W}_{y}x+{B}_{y},\end{array}$$

where ${W}_{x}$, ${B}_{x}$, ${W}_{y}$, and ${B}_{y}$ could be calculated using Eqs. (4) and (5), as follows (Fig. 5).

10 $$W_x={\textstyle\sum_{k=1}^n}M_kX_k$$

11 $$B_x={\textstyle\sum_{k=1}^n}N_kX_k$$

12 $$W_y={\textstyle\sum_{k=1}^n}M_kY_k$$

13 $$B_y={\textstyle\sum_{k=1}^n}N_kY_k$$

$$(M_k=\frac{nt_k-\sum_{l=1}^nt_l}C,N_k=\frac{\sum_{l=1}^n\left(t_l\right)^2-t_k\sum_{l=1}^nt_l}C,C=n{\textstyle\sum_{k=1}^n}\left(t_k\right)^2-\left({\textstyle\sum_{k=1}^n}t_k\right)^2)$$

Their expected values were as follows.

14 $$E\left(W_x\right)={\textstyle\sum_{k=1}^n}M_kE\left(X_k\right).$$

15 $$E\left(B_x\right)={\textstyle\sum_{k=1}^n}N_kE\left(X_k\right).$$

16 $$E\left(W_y\right)={\textstyle\sum_{k=1}^n}M_kE\left(Y_k\right).$$

17 $$E\left(B_y\right)={\textstyle\sum_{k=1}^n}N_kE\left(Y_k\right).$$

Therefore, we could conclude that the novel method was accurate when these expected values were consistent with the predetermined true values, *w*_*x*_, *b*_*x*_, *w*_*y*_, and *b*_*y*_, respectively.

## Results

### Conventional method

Because $\sqrt{{\left({X}_{k}\right)}^{2}+{\left({Y}_{k}\right)}^{2}}$ cannot be directly presented by a formula using $\sqrt{{\left({x}_{k}\right)}^{2}+{\left({y}_{k}\right)}^{2}}$ [4], we used the Taylor expansion to find the relationship between them. When *f* was defined as $f\left(x,y\right)=\sqrt{{x}^{2}+{y}^{2}}$, it could be Taylor-expanded around ${x}_{k}$ and ${y}_{k}$ as.

$$f\left({X}_{k},{Y}_{k}\right)=f\left({x}_{k},{y}_{k}\right)+\left[\left({x\mathrm{^{\prime}}}_{k}-{x\mathrm{^{\prime}}}_{0}\right)\frac{\partial }{\partial x}+\left({y\mathrm{^{\prime}}}_{k}-{y\mathrm{^{\prime}}}_{0}\right)\frac{\partial }{\partial y}\right]f\left({x}_{k},{y}_{k}\right)+\frac{1}{2}\left[{\left({x\mathrm{^{\prime}}}_{k}-{x\mathrm{^{\prime}}}_{0}\right)}^{2}\frac{{\partial }^{2}}{\partial {x}^{2}}+2\left({x\mathrm{^{\prime}}}_{k}-{x\mathrm{^{\prime}}}_{0}\right)\left({y\mathrm{^{\prime}}}_{k}-{y\mathrm{^{\prime}}}_{0}\right)\frac{{\partial }^{2}}{\partial x\partial y}+{\left({y\mathrm{^{\prime}}}_{k}-{y\mathrm{^{\prime}}}_{0}\right)}^{2}\frac{{\partial }^{2}}{\partial {y}^{2}}\right]f\left({x}_{k},{y}_{k}\right)+{R}_{n}.$$

When the expected values were considered,

$$E\left(\sqrt{{\left({X}_{k}\right)}^{2}+{\left({Y}_{k}\right)}^{2}}\right)=E\left(\sqrt{{\left({x}_{k}\right)}^{2}+{\left({y}_{k}\right)}^{2}}\right)+E\left(\left[\left({x\mathrm{^{\prime}}}_{k}-{x\mathrm{^{\prime}}}_{0}\right)\frac{\partial }{\partial x}+\left({y\mathrm{^{\prime}}}_{k}-{y\mathrm{^{\prime}}}_{0}\right)\frac{\partial }{\partial y}\right]f\left({x}_{k},{y}_{k}\right)\right)+E\left(\frac{1}{2}\left[{\left({x\mathrm{^{\prime}}}_{k}-{x\mathrm{^{\prime}}}_{0}\right)}^{2}\frac{{\partial }^{2}}{\partial {x}^{2}}+2\left({x\mathrm{^{\prime}}}_{k}-{x\mathrm{^{\prime}}}_{0}\right)\left({y\mathrm{^{\prime}}}_{k}-{y\mathrm{^{\prime}}}_{0}\right)\frac{{\partial }^{2}}{\partial x\partial y}+{\left({y\mathrm{^{\prime}}}_{k}-{y\mathrm{^{\prime}}}_{0}\right)}^{2}\frac{{\partial }^{2}}{\partial {y}^{2}}\right]f\left({x}_{k},{y}_{k}\right)\right)+E\left({R}_{n}\right).$$

When Eq. (2) was assigned, $x'_k$ and $y'_k$ were independent and $E\left({R}_{n}\right)$ was approximated as zero:

$$E\left(\sqrt{{\left({X}_{k}\right)}^{2}+{\left({Y}_{k}\right)}^{2}}\right)\approx \sqrt{{\left({x}_{k}\right)}^{2}+{\left({y}_{k}\right)}^{2}}+E\left(\frac{1}{2}\left[{\left({{x}^{\mathrm{^{\prime}}}}_{k}-{{x}^{\mathrm{^{\prime}}}}_{0}\right)}^{2}\frac{{\partial }^{2}}{\partial {x}^{2}}+{\left({{y}^{\mathrm{^{\prime}}}}_{k}-{{y}^{\mathrm{^{\prime}}}}_{0}\right)}^{2}\frac{{\partial }^{2}}{\partial {y}^{2}}\right]f\left({x}_{k},{y}_{k}\right)\right).$$

Because the variance of $x'_k$ and  could be considered equivalent ($\left({\sigma}_{x_k^{\prime }}={\sigma}_{y_k^{\prime }}={\sigma}_e\right)$, where $\sigma$ denotes the standard deviation),

18 $$E\left(\sqrt{{\left({X}_{k}\right)}^{2}+{\left({Y}_{k}\right)}^{2}}\right)=\sqrt{{\left({x}_{k}\right)}^{2}+{\left({y}_{k}\right)}^{2}}+\frac{{\left[{\sigma }_{\left({x\mathrm{^{\prime}}}_{k}-{x\mathrm{^{\prime}}}_{0}\right)}\right]}^{2}{\left({y}_{k}\right)}^{2}+{\left[{\sigma }_{\left({y\mathrm{^{\prime}}}_{k}-{y\mathrm{^{\prime}}}_{0}\right)}\right]}^{2}{\left({x}_{k}\right)}^{2}}{2{\sqrt{{\left({x}_{k}\right)}^{2}+{\left({y}_{k}\right)}^{2}}}^{3}}=\sqrt{{\left({x}_{k}\right)}^{2}+{\left({y}_{k}\right)}^{2}}+\frac{{\left({\sigma }_{e}\right)}^{2}}{\sqrt{{\left({x}_{k}\right)}^{2}+{\left({y}_{k}\right)}^{2}}}.$$

(See Additional file 1: Appendix (1).)

Because $E\left(sgn\left({Y}_{k}\right)\right)$ can vary between 0 and 1 according to the value of ${y}_{k}$, ${y}_k^{\prime}$, and ${y}_0^{\prime }$, $E\left({P}_{k}\right)$ could vary between 0 and $\sqrt{{\left({x}_{k}\right)}^{2}+{\left({y}_{k}\right)}^{2}}+\frac{{\left({\sigma }_{e}\right)}^{2}}{\sqrt{{\left({x}_{k}\right)}^{2}+{\left({y}_{k}\right)}^{2}}}$. Therefore, Eq. (9) could not always be satisfied.

### Novel method

According to the definitions of ${X}_{k}$ and ${Y}_{k}$ (Eq. (3)),

$$\begin{array}{c}E\left({X}_{k}\right)=E({x}_{k}+{x\mathrm{^{\prime}}}_{k}-{x\mathrm{^{\prime}}}_{0})={x}_{k}\\ E\left({Y}_{k}\right)=E\left({y}_{k}+{y\mathrm{^{\prime}}}_{k}-{y\mathrm{^{\prime}}}_{0}\right)={y}_{k}.\end{array}$$

Therefore, Eqs. (14)–(17) could be transformed into

19 $$E\left(W_x\right)={\textstyle\sum_{k=1}^n}M_kx_k.$$

20 $$E\left(B_x\right)={\textstyle\sum_{k=1}^n}N_kx_k.$$

21 $$E\left(W_y\right)={\textstyle\sum_{k=1}^n}M_ky_k.$$

22 $$E\left(B_y\right)={\textstyle\sum_{k=1}^n}N_ky_k.$$

From the combination of Eqs. (19) and (20), we know that the best-fit line for points $\left({t}_{k},{x}_{k}\right)$ (*k* = 1, 2, …, *n*) is.

$$y=E\left({W}_{x}\right)x+E\left({B}_{x}\right).$$

When Eq. (1) is taken into account,

$$E\left({W}_{x}\right)={w}_{x}$$

$$E\left({B}_{x}\right)={b}_{x}.$$

From the combination of Eqs. (21) and (22), we know that the best-fit line for points $\left({t}_{k},{y}_{k}\right)$ (*k* = 1, 2, …, *n*) is.

$$y= E\left({W}_{y}\right)x+E\left({B}_{y}\right).$$

Similarly,

$$E\left({W}_{y}\right)={w}_{y}$$

$$E\left({B}_{y}\right)={b}_{y}.$$

These results demonstrate the accuracy of the novel method.

## Discussion

This study mathematically demonstrates both the insufficient accuracy of the conventional method and the sufficient accuracy of the novel method for wear calculation. Insufficient accuracy of the conventional method was proved by the fact that Eq. (9) was not always satisfied. Given the same reason, the conventional calculation could not be sufficiently accurate even if the other option to treat the negative wear (assuming zero or neglecting) was adopted. Discrepancy between the average vector ($\sqrt{{\left({x}_{k}\right)}^{2}+{\left({y}_{k}\right)}^{2}}$) and the average scalar ($E\left(\sqrt{{\left({X}_{k}\right)}^{2}+{\left({Y}_{k}\right)}^{2}}\right)$) was also discussed in other fields of study. Ranacher et al. demonstrated that the distance between two points recorded with the Global Positioning System (GPS) is, on average, larger than the true distance [24]. This discrepancy is due in part to the uncertainty in location measurement by the GPS and results in a difference between the average vector and scalar lengths. Similar findings have been reported in the field of wind speed measurement [25]. Thus, transformation of vector measurement data into scalar before averaging has a potential to lead to wrong calculation results.

The latest measurement techniques, such as dual circle and radiostereometric analysis, provides penetration data in vector format, but they have been then usually transformed into scalar by wear measurement applications for the purpose of applying the conventional calculation method. However, we demonstrated more accuracy with the novel method, which uses vector penetration data intact. We recommend wear researchers to adopt the novel method in the wear analysis, and, simultaneously, manufacturers of the wear measurement application to provide the penetration data in the vector format as the result of measurement.

The accuracy of the novel method was evaluated in the situation of wear calculation of a single hip. Practically, we can use spreadsheet software, such as Excel (Microsoft Corp., Redmond, WA, USA), for the calculation using the novel method. There are several ways to perform the calculation on Excel after collecting all head center penetration vector data throughout the postoperative period. We can enter the formulae (Eqs. (10)–(13)) into a spreadsheet for direct calculations, generate an approximate straight line in a scatterplot, or use “Solver” functions to calculate $\left({B}_{x},{B}_{y}\right)$ and $\left({W}_{x},{W}_{y}\right).$ For better comprehending the novel method, an example for calculating the bedding-in and wear rate are presented in Additional file 1: Appendix (2). Furthermore, we can use the mean and standard deviation of each component to statistically summarize the data of a group of hips as: $\left(\overline{{B }_{x}}\pm {\sigma }_{{B}_{x}},\overline{{B }_{y}}\pm {\sigma }_{{B}_{y}}\right)$ and $\left(\overline{{W }_{x}}\pm {\sigma }_{{W}_{x}},\overline{{W }_{y}}\pm {\sigma }_{{W}_{y}}\right).$ Regarding the clinical significance of wear data, using average penetration $\sqrt{{\left(\overline{{W }_{x}}\right)}^{2}+{\left(\overline{{W }_{y}}\right)}^{2}}\pm \sqrt{\frac{{\left(\overline{{W }_{x}}\right)}^{2}{\left({\sigma }_{{W}_{x}}\right)}^{2}+{\left(\overline{{W }_{y}}\right)}^{2}{\left({\sigma }_{{W}_{y}}\right)}^{2}}{{\left(\overline{{W }_{x}}\right)}^{2}+{\left(\overline{{W }_{y}}\right)}^{2}}}$ (Additional file 1: Appendix (3) [26]) may also be suitable to compare between groups.

When the linear wear rate was provided in the vector format, how should the volumetric wear rate be calculated? There are several popular methods for the volumetric wear approximate calculation [27, 28]. Wear depth (*d*) and wear angle (*β*) are unexceptionally necessary for them, and the former is a scalar value transformed from the penetration vector. Therefore, it should be avoided to average or apply the least-squares method to values of the volumetric wear if possible. We consider that it is more accurate to calculate the volumetric wear rate of a single hip using the linear wear rate vector obtained by the novel method than by applying the least-squares method to multiple values of the volumetric wear obtained for each follow-up period. We also consider that to use the average wear vector to calculate the average volumetric wear of a group of hips would be more accurate than to average values of volumetric wear obtained for multiple cases. When *W*′*x* and *W*′*y* were defined as the components of the wear vector calibrated by the cup inclination angle, $\sqrt{{\left(\overline{{W }_{x}}\right)}^{2}+{\left(\overline{{W }_{y}}\right)}^{2}}\left(=\sqrt{\left({\overline{W^\prime_x}}\right)^2+\left({\overline{W^\prime_y}}\right)^2}\right)$ can be used for average *d*. When $\overline{W^\prime_x}\ge0$, ${\tan}^{-1}\frac{\overline{W^\prime_y}}{\overline{W^\prime_x}}$ can be used for *β*, and when $\overline{W^\prime_x}<0$, ${\tan}^{-1}\frac{\overline{W^\prime_y}}{\overline{W^\prime_x}}+\pi$ can be used [29].

There is a limitation to this study. The wear progresses 3-dimensionally in vivo, although this study evaluated the accuracy of only 2-dimensional wear calculations. We consider that the conventional method would also be less accurate, and the novel method would be more accurate when they were applied to 3-dimensional wear calculations because a *z* component could be added to the formulae without impairing solvability. These were simply testified in Additional file 1:  Appendix (4).

## Supplementary Information

**Additional file 1. **

## Data Availability

The datasets generated and analyzed during the current study are available from the corresponding author on reasonable request.
