# Supplementary material for: The superior accuracy of a novel method in total hip wear calculations following radiographic measurement
Source: BMC Musculoskelet Disord. 2022 Feb 9;23:130. doi: 10.1186/s12891-021-04964-5 (PMC8826684; doi:10.1186/s12891-021-04964-5)
Supplement: Supplementary file 1 — Additional file 1. [file 12891_2021_4964_MOESM1_ESM.docx]

**Appendix (1)**

When *f* is defined as $f\left( x,y \right)=\sqrt{x^{2}+y^{2}}$, partial differentiation provides:

$\frac{\partial}{\partial x}f\left( x,y \right)=\frac{x}{\sqrt{x^{2}+y^{2}}}$

$\frac{\partial}{\partial y}f\left( x,y \right)=\frac{y}{\sqrt{x^{2}+y^{2}}}$

$\frac{\partial^{2}}{\partial x^{2}}f\left( x,y \right)=\frac{y^{2}}{\left( \sqrt{x^{2}+y^{2}} \right)^{3}}$

$\frac{\partial^{2}}{\partial y^{2}}f\left( x,y \right)=\frac{x^{2}}{\left( \sqrt{x^{2}+y^{2}} \right)^{3}}$.

**Appendix (2)**

First, the head center coordinates of a hip are assumed to be measured as follows at each period.

$\left( x_{0}+{x'}_{0},y_{0}+{y'}_{0} \right)=\left( -0.07, 0.18 \right)$, t_0_ = 0

$\left( x_{1}+{x'}_{1},y_{1}+{y'}_{1} \right)=\left( 0.12, 0.16 \right)$, t_1_ = 2.1

$\left( x_{2}+{x'}_{2},y_{2}+{y'}_{2} \right)=\left( 0.03, 0.22 \right)$, t_2_ = 4.3

$\left( x_{3}+{x'}_{3},y_{3}+{y'}_{3} \right)=\left( 0.18, 0.27 \right)$, t_3_ = 6

$\left( x_{4}+{x'}_{4},y_{4}+{y'}_{4} \right)=\left( 0.1, 0.51 \right)$, t_4_ = 8.2

$\left( x_{5}+{x'}_{5},y_{5}+{y'}_{5} \right)=\left( 0.25, 0.32 \right)$, t_5_ = 9.6

(*n* = 5).

Then, $\left( X_{1},Y_{1} \right)=\left( 0.19, -0.02 \right)$, $\left( X_{2},Y_{2} \right)=\left( 0.1, 0.04 \right)$, $\left( X_{3},Y_{3} \right)=\left( 0.25, 0.09 \right)$, $\left( X_{4},Y_{4} \right)=\left( 0.17, 0.33 \right)$, $\left( X_{5},Y_{5} \right)=\left( 0.32, 0.14 \right)$. We can enter these data and the formulae (Eqs. (10)–(13)) into a spreadsheet for direct calculations by the novel method.

$W_{x}=\frac{\left. 5\sum_{k=1}^{5} t_{k}X_{k}-\sum_{k=1}^{5} t_{k}\sum_{k=1}^{5} X_{k} \right.}{C}\approx0.016$

$W_{y}=\frac{\left. 5\sum_{k=1}^{5} t_{k}Y_{k}-\sum_{k=1}^{5} t_{k}\sum_{k=1}^{5} Y \right.}{C}\approx0.034$

$B_{x}=\frac{\left. \sum_{k=1}^{5} \left( t_{k} \right)^{2}\sum_{k=1}^{5} X_{k}-\sum_{k=1}^{5} t_{k}X_{k}\sum_{k=1}^{5} t_{k} \right.}{C}\approx0.109$

$B_{y}=\frac{\left. \sum_{k=1}^{5} \left( t_{k} \right)^{2}\sum_{k=1}^{5} Y_{k}-\sum_{k=1}^{5} t_{k}Y_{k}\sum_{k=1}^{5} t_{k} \right.}{C}\approx-0.089$

($C=5\sum_{k=1}^{5} {(t_{k})}^{2}-\left( \sum_{k=1}^{5} t_{k} \right)^{2}=179.460$).

Thus, the wear rate and bedding-in of this hip are (0.016, 0.034) and (0.109, -0.089), respectively, when the data were rounded off to the third decimal place.

These vectors can also be obtained by generating an approximate straight line in a scatterplot.


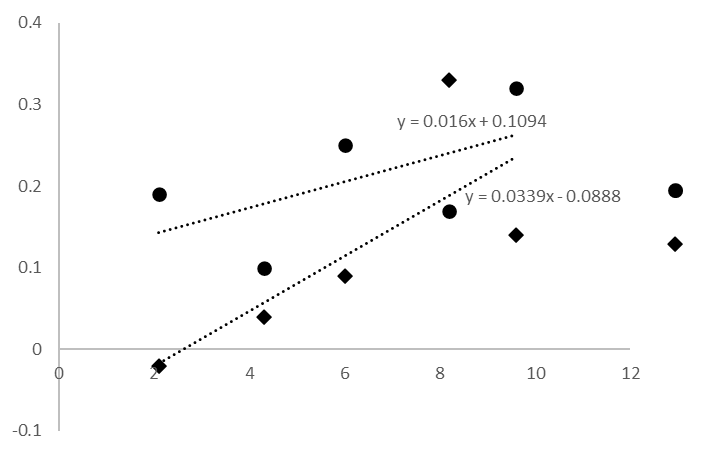


*X_k_*

*Y_k_*

**Appendix (3)**

When $z=f\left( x,y \right)$ and $x=x_{0}\pm\sigma_{x}$, $y=y_{0}\pm\sigma_{y}$, $z=z_{0}\pm\sigma_{z}$, then $z_{0}=f\left( x_{0},y_{0} \right)$ and $\sigma_{z}=\sqrt{\left( \frac{\partial f}{\partial x} \right)^{2}\left( \sigma_{x} \right)^{2}+\left( \frac{\partial f}{\partial y} \right)^{2}\left( \sigma_{y} \right)^{2}}$, according to the law of error propagation [26]. When $f\left( x,y \right)=\sqrt{x^{2}+y^{2}}$ (as in Appendix (1)), the standard deviation of *z* is calculated as

$\sigma_{z}=\sqrt{\frac{{x_{0}}^{2}\left( \sigma_{x} \right)^{2}+{y_{0}}^{2}\left( \sigma_{y} \right)^{2}}{{x_{0}}^{2}+{y_{0}}^{2}}}$.

**Appendix (4)**

When the *z* component was added in the definitions and assumptions, Eqs. (6) and (9) could be substituted with Eqs. (23) and (24), and Eqs. (25) and (26) should be added to Eqs. (14)–(17).

$P_{k}=sgn\left( Y_{k} \right)\sqrt{\left( X_{k} \right)^{2}+\left( Y_{k} \right)^{2}+\left( Z_{k} \right)^{2}}$, (23)

$E\left( P_{k} \right)=\sqrt{\left( x_{k} \right)^{2}+\left( y_{k} \right)^{2}+\left( z_{k} \right)^{2}}$ (24)

$E\left( W_{z} \right)=\sum_{k=1}^{n} M_{k}E\left( Z_{k} \right)$ (25)

$E\left( B_{z} \right)=\sum_{k=1}^{n} N_{k}E\left( Z_{k} \right)$ (26)

The following approximation could be derived:

$E\left( \sqrt{\left( X_{k} \right)^{2}+\left( Y_{k} \right)^{2}+\left( Z_{k} \right)^{2}} \right)\approx\sqrt{\left( x_{k} \right)^{2}+\left( y_{k} \right)^{2}+\left( z_{k} \right)^{2}}+\frac{\left( \sigma_{e} \right)^{2}}{\sqrt{\left( x_{k} \right)^{2}+\left( y_{k} \right)^{2}+\left( z_{k} \right)^{2}}}$.

Therefore, the conventional method was less accurate in the 3-dimensional conditions.

Alternatively, Eqs. (25) and (26) could be transformed into

$E\left( W_{z} \right)=\sum_{k=1}^{n} M_{k}z_{k}$

$E\left( B_{z} \right)=\sum_{k=1}^{n} N_{k}z_{k}$.

Therefore,

$E\left( W_{z} \right)=w_{z}$

$E\left( B_{z} \right)=b_{z}$.

Because the addition of the *z* component did not impair the accuracy of the calculations of *x* and *y* components, the novel method was accurate in the 3-dimensional conditions.
